# Searching for responders to multidomain dementia prevention in late life: A pooled analysis of individual participant data from the MAPT and preDIVA trials

**DOI:** 10.1002/alz.14472

**Published:** 2025-01-17

**Authors:** Nicola Coley, Marieke P. Hoevenaar‐Blom, Jason Shourick, Eric P. Moll van Charante, Jan‐Willem van Dalen, Willem A. van Gool, Edo Richard, Sandrine Andrieu, the preDIVA study group and the MAPT/IHU HealthAge Open Science group

**Affiliations:** ^1^ Aging Research Team Centre for Epidemiology and Research in Population health (CERPOP) INSERM‐University of Toulouse UPS Toulouse France; ^2^ Department of Epidemiology and Public Health Toulouse University Hospital Toulouse France; ^3^ IHU HealthAge, Cité de la santé Toulouse France; ^4^ Department of General Practice Amsterdam UMC, Location AMC Amsterdam the Netherlands; ^5^ Department of Public and Occupational Health Amsterdam UMC, Location VUMC Amsterdam the Netherlands; ^6^ Department of Neurology Donders Centre for Brain Behaviour and Cognition Radboud University Medical Centre Nijmegen the Netherlands; ^7^ Department of Neurology Amsterdam UMC University of Amsterdam Amsterdam the Netherlands

**Keywords:** dementia, differential intervention effect, efficacy, multidomain intervention, prevention, risk, subgroup analysis

## Abstract

**INTRODUCTION:**

It is unknown in which, if any, subgroups of older adults multidomain interventions are effective at reducing long‐term dementia incidence.

**METHODS:**

We pooled up to 12 years of follow‐up data from 5205 participants aged > 70 from the Multidomain Alzheimer Preventive Trial (MAPT) and Prevention of Dementia by Intensive Vascular Care (preDIVA) studies. The primary outcome was incident all‐cause dementia. Pre‐specified subgroups were defined by dementia risk factors (age, sex, education, apolipoprotein E [*APOE*] genotype, cognitive status, and cardiovascular risk factors).

**RESULTS:**

Four hundred eighty‐six participants developed dementia during 37,782 person‐years of follow‐up. Higher incidence was associated with baseline age, *APOE* ε4 genotype, physical inactivity, Mini‐Mental State Examination, and blood pressure. Multidomain intervention had no effect on incident dementia overall (hazard ratio = 0.98, 95% confidence interval 0.80–1.21), or in any pre‐specified subgroup. A recursive partitioning algorithm also did not detect any subgroups, defined by single or multiple risk factors, showing a differential intervention effect.

**DISCUSSION:**

We did not identify any subgroups of older adults in whom multidomain interventions significantly reduced incident dementia.

**CLINICAL TRIAL REGISTRATION:**

MAPT: NCT00672685 (clinicaltrials.gov); PreDIVA: ISRCTN29711771 (ISRCTN registry)

**Highlights:**

- We pooled up to 12 years of follow‐up data from two multidomain prevention trials.
- Five thousand two hundred five participants aged ≥ 70 were included.
- Subgroups were pre‐defined by modifiable and non‐modifiable dementia risk factors.
- A data‐driven recursive partitioning algorithm was also used.
- Multidomain intervention did not lower incident dementia overall or in any subgroup.

## 1 BACKGROUND

Modifiable risk factors, including lifestyle and cardiovascular risk factors, are estimated to underlie up to 45% of dementia cases.^1^ Multidomain interventions, targeting several modifiable risk factors simultaneously, are considered particularly promising for dementia prevention,^2^, ^3^ but randomized controlled trials (RCTs) have so far provided limited evidence of their effects on dementia or cognitive decline, and follow‐up periods have generally been relatively short.^4^, ^5^, ^6^, ^7^, ^8^, ^9^, ^10^

One factor which could explain the mostly neutral trial results is that these interventions may only be effective in certain specific subgroups of individuals. Indeed, existing evidence points to greater efficacy in those with increased dementia risk or with a sufficient window of opportunity for risk profile improvement.^10^ For example, the Finnish Geriatric Intervention Study to Prevent Cognitive Impairment and Disability (FINGER) study's primary analysis showed improved cognition after a 2‐year multidomain intervention in individuals with an increased CAIDE (Cardiovascular Risk Factors, Aging, and Incidence of Dementia) dementia risk score,^6^ and similar results were found in a Multidomain Alzheimer Preventive Trial (MAPT) subgroup analysis.^11^ In addition, Rovner et al. found beneficial effects of a 2‐year multidomain intervention in individuals with mild cognitive impairment (MCI) in their primary analysis.^7^ Trends from subgroup analyses, which tend to be underpowered, also suggest that multidomain interventions might lower cognitive decline or dementia incidence in apolipoprotein E (*APOE*) ε4 carriers, a well‐established genetic risk factor for late onset Alzheimer's disease (AD), or in individuals with untreated hypertension.^4^, ^5^, ^12^, ^13^, ^14^ Additionally, another multidomain prevention trial showed a stronger intervention effect on modifiable dementia risk factors in those with lower, compared to higher, education.^15^ Thus, a more tailored approach to multidomain dementia prevention, targeting interventions to those most likely to benefit from them, could be more effective than a one‐size‐fits‐all approach applied to general/unselected populations.

Previous studies assessing multidomain intervention efficacy in specific subgroups have mostly been restricted either to individual trials rather than pooled or meta‐analyses,^4^, ^5^, ^6^, ^8^ thus limiting statistical power, or to limited follow‐up periods,^16^ and have focused mainly on cognitive function outcomes rather than incident dementia.^17^ Whether the efficacy of multidomain interventions for reducing long‐term dementia incidence differs across subgroups of older people is as yet unknown.

The aim of this study was therefore to combine data from two of the largest multidomain dementia prevention RCTs with long‐term follow‐up, to explore whether multidomain interventions may be effective in reducing dementia incidence in certain subgroups of older adults, defined by dementia risk factors. We hypothesized that in adults aged ≥ 70, multidomain interventions are more effective for reducing the risk of all‐cause and AD dementia, and cognitive decline, in high‐risk subgroups defined by risk factors including high body mass index (BMI), cholesterol, and blood pressure; limited physical activity; low education; and/or an unfavorable *APOE* ε4 genotype (ε4 hetero‐ or homozygotes), and are less effective at older ages.

## 2 METHODS

### 2.1 Participants and setting

We combined individual participant data from the randomized controlled MAPT and Prevention of Dementia by Intensive Vascular Care (preDIVA) prevention trials, including their extended observational follow‐up periods. Both have been fully described previously.^4^, ^5^ Briefly, MAPT tested a 3‐year multidomain intervention (group‐based sessions including cognitive training and education about physical activity and a healthy diet; and an annual prevention consultation targeting cardiovascular/dementia risk factors), omega‐3 fatty acid supplementation (800 mg docosahexaenoic acid and 225 mg eicosapentaenoic acid daily), or both interventions combined, compared to placebo, for the prevention of cognitive decline. In the present analysis, we compared individuals who received the multidomain intervention to those who did not, regardless of omega‐3 assignment, to focus on multidomain interventions as there was no significant effect of omega‐3 supplementation alone in the primary MAPT analysis or in any pre‐specified subgroups.^4^ MAPT participants were 1679 dementia‐free (verified at baseline using Diagnostic and Statistical Manual of Mental Disorders Fourth Edition [DSM‐IV] criteria) community‐dwelling individuals aged ≥ 70 with subjective memory complaints, at least one instrumental activity of daily living limitation, and/or slow gait speed. At baseline, they had a Mini‐Mental State Examination (MMSE)^18^ score ≥ 24, and no difficulties with basic activities of daily living. Recruitment took place between May 2008 and February 2011 in 13 memory centers in France and Monaco. After the 3‐year intervention period, participants were invited to participate in a 2‐year observational extension period.

**RESEARCH IN CONTEXT**

1. **Systematic review**: We performed a literature search using PubMed. Randomized controlled trials (RCTs) have provided little evidence of a protective effect of multidomain interventions on cognitive decline or dementia in the general population of older adults, but there have been suggestions, mostly based on single studies targeting cognitive decline, of greater efficacy in subgroups with increased dementia risk or with a sufficient window of opportunity for risk profile improvement.
2. **Interpretation**: We did not identify any subgroups of older adults in whom multidomain interventions significantly reduced incident dementia. Considering our results and those of other RCTs, the potential impact of multidomain interventions for dementia prevention at the individual level, even among those at greatest risk of dementia, appears modest at best.
3. **Future directions**: Given the inherent limitations of RCTs, population‐level public health approaches to dementia prevention might be a useful complementary strategy.

PreDIVA tested a 6‐ to 8‐year multidomain nurse‐led vascular care intervention (individual 4 monthly visits involving assessment of cardiovascular risk factors [diet, physical activity, weight, smoking, and blood pressure] and tailored lifestyle and medication advice), versus usual care according to the prevailing standards for cardiovascular risk management, for the prevention of dementia in 3526 dementia‐free individuals aged 70 to 78 at baseline in the Netherlands. Participants were recruited via 116 general practices within 26 health‐care centers between June 2006 and March 2009. The trial was cluster‐randomized, with general practices as the unit of randomization. After the initial 6‐ to 8‐year intervention period, dementia status was further assessed up to 10 to 12 years post‐baseline in all surviving participants who were dementia free (*n* = 2708 [77%]) during the intervention phase.^19^

Neither trial demonstrated a significant effect of the multidomain intervention on the pre‐specified primary outcome measure in the primary analysis, but subgroup analyses indicated evidence of beneficial effects in some at‐risk groups defined by an elevated CAIDE dementia risk score,^4^ evidence of positron emission tomography amyloid pathology,^4^ or untreated hypertension at baseline.^5^

MAPT was approved by the Toulouse Ethics Committee (CPP SOOM II) and the French Health Authority. PreDIVA was approved by the medical ethics committee of the Academic Medical Centre, Amsterdam, the Netherlands. All participants provided written informed consent. The trials are registered at ClinicalTrials.gov (MAPT: NCT00672685) and ISRCTN (preDIVA: ISRCTN29711771).

### 2.2 Outcome measures

The primary outcome for the present analyses was incident all‐cause dementia.

In MAPT, dementia was a secondary outcome, and was diagnosed by study investigators in hospital memory centers based on clinical criteria, following standard practice in each of the participating memory centers (DSM‐IV criteria were used at the time of the study). Dementia diagnoses and probable etiology were reviewed first by an internal study committee, and then by an independent external committee, both of which had access to the participants’ cognitive evaluations (a full battery of cognitive tests was performed every 6 months for the first year of follow‐up, and annually thereafter during both the intervention period and the extended observational follow‐up period).^4^

In preDIVA, dementia was the primary outcome and was diagnosed according to DSM‐IV criteria. Outcome data were collected during follow‐up visits, and by telephone (the Telephone Interview of Cognitive Status was used to screen for cognitive decline) for the observational follow‐up period, supplemented with information from general practitioner (GP) electronic health records and the Dutch National Death Registry.^5^, ^19^ An independent outcome adjudication committee adjudicated all clinical outcomes.^5^

Secondary outcomes were incident AD dementia, and cognitive decline, as measured by the MMSE (at baseline and months 6, 12, 24, 36, 48, and 60 in MAPT, and at baseline, and months 24, 48, and 60 to 84 [i.e., at the end of the intervention period] in preDIVA).^18^

### 2.3 Subgroups

Subgroups were pre‐specified for the current analysis, and defined by the following baseline characteristics, which were expected to be associated with dementia risk: age (< 75 vs. ≥ 75 years), sex (male vs. female), level of education (low [primary school certificate or lower], medium [middle/vocational school], or high [high school diploma (e.g., baccalaureate) or higher]), *APOE* genotype (ε4 non‐carrier vs. ε4 carrier), cognitive status (MMSE ≥ 26 vs. < 26), BMI (≤ 30 vs. > 30), total cholesterol (≤ 6.5 mmol/L vs. > 6.5 mmol/L), physical activity (≥ 150 minutes/week vs. < 150 minutes/week), systolic blood pressure (SBP; ≤ 140 mmHg vs. > 140 mmHg), untreated hypertension (no vs. yes), and CAIDE dementia risk score (< 6 vs. ≥ 6 points).

In addition to these pre‐specified subgroups, we performed an exploratory, data‐driven analysis aiming to identify subgroups with differential intervention effects, using a search algorithm explained below. These subgroups could be defined by one or more of the risk factors.

### 2.4 Statistical analysis

All analyses were conducted on available data following the intention to treat principle. Baseline characteristics are presented as mean (standard deviation [SD]) or median (interquartile range) for continuous variables and frequency (percentage) for categorical variables. Between‐group comparisons of baseline characteristics were performed, as applicable, using *t* tests or Wilcoxon tests for continuous variables, and chi^2^ tests for categorical variables.

The primary analysis was performed using shared frailty Cox proportional hazards models with a random intercept for study center (MAPT) or GP practice (preDIVA), to take into account within‐trial and ‐center clustering.^20^ One model was run for each subgroup, including all participants with available data for the subgroup of interest at baseline. Time to event was defined as the time from randomization to dementia diagnosis, and between‐subgroup differences in intervention effects were assessed by subgroup × intervention group interaction terms. Participants who were not diagnosed with dementia were censored at the date of their last study visit in MAPT and at the date of telephone assessment or retrieval of data from the GP registries in preDIVA.

Results are reported as hazard ratios (HRs) and 95% confidence intervals (95% CIs). Models were first run unadjusted and then with adjustment for variables showing baseline imbalances between the pooled multidomain and control groups. Kaplan–Meier survival curves were also plotted. Similar models were used to assess intervention effects on the secondary outcome of AD dementia, excluding participants who were diagnosed with dementia and for whom the probable etiology was unknown (*n* = 191).

Longitudinal mixed effects models were used to analyze change in MMSE scores over time in participants with at least two MMSE measures. Follow‐up time was included as a continuous variable, and the rate of change was linear (quadratic and cubic time terms were tested and found to be non‐significant in both trials, and in the pooled dataset). Models included a three‐way subgroup × intervention × time interaction (to assess between‐subgroup differences in intervention effects—these were considered significant if the *P* value for the interaction term was < 0.05) in the fixed effects; random intercepts at the participant, center, and trial level; and a random slope for time at the individual level. Such models use all available data points, and assume missing data are missing at random. Mean between‐randomization group differences (and their 95% CIs) in the annual rate of MMSE change were calculated for each subgroup using the stata lincom post‐estimation command.

Finally, we performed an exploratory data‐driven analysis aiming to identify subgroups that could be defined (by a data‐driven algorithm) using single or combinations of risk factors, with differential intervention effects on incident dementia. For this, we used a recursive partitioning procedure based on the interaction between a given subgroup and the intervention effect, the SIDES (subgroup identification based on differential effect search) method.^21^ The SIDES method was optimized to identify any group of > 100 subjects in which there was any differential intervention effect compared to the rest of the population. All characteristics used to define the pre‐specified subgroups in the primary analysis were included as potential partitioning variables in the SIDES analysis, and the depth (meaning the number of variables that the method could use to define a group) was set to 17, meaning that potentially all variables could be conjointly used to define a subgroup. Of note, as the SIDES method can be used to define cut‐offs, we included quantitative variables as continuous variables without dichotomizing them a priori. The SIDES analysis was performed on the population with complete data (*n* = 3786) and on the whole population after imputation of missing data using the non‐parametric random forest method (R “MissForest” package; *n* = 5205)

All analyses were performed in Stata version 18.0 (StataCorp LP]), except for the recursive partitioning analysis, which was performed in R version 4.2.2. Given the exploratory nature of this study, we did not adjust for multiple statistical comparisons.

## 3 RESULTS

The baseline characteristics of the 5205 participants in the pooled dataset are shown in Table 1. Compared to MAPT participants, preDIVA participants were significantly younger, had significantly higher mean CAIDE dementia risk scores; BMI, and SBP; more often had a history of diabetes, stroke, and heart disease; and were more likely to be male, be *APOE* ε4 carriers, and have untreated hypertension. They also had a significantly lower level of education, had lower cholesterol, and were more likely to be physically active. Despite the differences in baseline characteristics between the two trials, the multidomain (i.e., receiving group‐based cognitive training, education about physical activity and a healthy diet, and an annual prevention consultation targeting cardiovascular/dementia risk factors in MAPT, and individual 4‐monthly visits with a nurse for assessment of cardiovascular risk factors and tailored lifestyle and medication advice in preDIVA) and control (i.e., usual care) groups in the pooled dataset were well balanced at baseline, except that SBP was slightly higher (mean 151.9 mmHg [SD 22.3] vs. 149.5 mmHg [21.3], *P* < 0.001) and low‐density lipoprotein (LDL) cholesterol slightly lower (mean 3.1 mmol/L [1.0] vs. 3.2 mmol/L [1.0], *P* = 0.024) in the intervention group, compared to the control group. MAPT participants who participated in the extended observational follow‐up were younger, had better cognitive performance, a lower CAIDE dementia risk score, a higher level of education, and were less likely to be diabetic or to be *APOE* ε4 carriers, than those who did not, but the participation rate did not differ by randomization group (Table S1 in supporting information).

**TABLE 1:** Baseline characteristics by (a) randomization group in the pooled dataset and (b) trial.

	Comparison by pooled randomization group			Comparison by trial		
	Control (*N *= 2478)	Intervention (*N* = 2727)	*P*	MAPT (*N* = 1679)	PreDIVA (*N* = 3526)	*P*
Age, median (IQR)	74 (72–77)	74 (72–76)	0.813	75 (72–78)	74 (72–76)	<0.001
MMSE, median (IQR)	28 (27–29)	28 (27–29)	0.585	28 (27–29) *[28.07]*	28 (27–29) *[28.12]*	0.040
CAIDE, mean (SD)	8.2 (1.9)	8.3 (1.9)	0.752	7.3 (1.9)	8.6 (1.8)	<0.001
BMI, mean (SD)	26.9 (4.1)	27.1 (4.2)	0.117	26.1 (4.1)	27.5 (4.2)	<0.001
SBP (mmHg), mean (SD)	149.5 (21.3)	151.9 (22.3)	<0.001	141.0 (19.7)	155.4 (21.3)	<0.001
LDL cholesterol (mmol/L), mean (SD)	3.2 (1.0)	3.1 (1.0)	0.024	3.3 (0.9)	3.1 (1.0)	<0.001
Total cholesterol (mmol/L), mean (SD)	5.4 (1.1)	5.3 (1.1)	0.093	5.7 (1.1)	5.2 (1.1)	<0.001
Female, *n* (%)	1427 (57.6%)	1579 (57.9%)	0.818	1087 (64.7%)	1919 (54.4%)	<0.001
Education, *n* (%)			0.905			<0.001
Low	430 (17.7%)	491 (18.1%)		85 (5.2%)	836 (24.0%)	
Medium	1332 (54.9%)	1485 (54.9%)		839 (51.1%)	1978 (56.7%)	
High	665 (27.4%)	731 (27.0%)		719 (43.8%)	577 (19.4%)	
Diabetes, *n* (%)	370 (14.9%)	491 (18.1%)	0.467	149 (8.9%)	648 (18.4%)	<0.001
Stroke, *n* (%)	219 (8.9%)	221 (8.2%)	0.354	93 (5.6%)	347 (10.0%)	<0.001
Heart disease, *n* (%)	569 (23.1%)	640 (23.6%)	0.689	165 (9.8%)	1044 (29.8%)	<0.001
≥150 minutes physical activity/week, *n* (%)	1956 (79.2%)	2104 (77.4%)	0.134	1211 (72.1%)	2849 (81.2%)	<0.001
*APOE* ε4, *n* (%)	513 (25.5%)	601 (26.7%)	0.357	299 (23.0%)	815 (27.5%)	0.002
Untreated hypertension	675 (27.3%)	794 (29.2%)	0.125	292 (17.4%)	1177 (33.5%)	<0.001

Abbreviation: *APOE*, apolipoprotein E; BMI, body mass index; CAIDE, Cardiovascular Risk Factors, Aging, and Incidence of Dementia; IQR, interquartile range; LDL, low‐density lipoprotein; MAPT, Multidomain Alzheimer Preventive Trial; MMSE, Mini‐Mental State Examination; preDIVA, Prevention of Dementia by Intensive Vascular Care; SBP, systolic blood pressure; SD, standard deviation.

In total, there were 486 cases of dementia (77/1679 [4.6%] in MAPT; 409/3491 [11.7%] in preDIVA) during a total follow‐up of 37,782 person‐years. The overall incidence of all‐cause dementia was 1.28/100 person‐years (MAPT: 1.25/100 person‐years; preDIVA 1.29/100 person‐years). Table 2 shows the incidence of dementia in the subgroups of interest. The risk of incident dementia was higher in older versus younger participants, in *APOE* ε4 carriers versus non‐carriers, in those with an MMSE < 26 at baseline versus those with a higher MMSE score, and in individuals who were physically inactive versus those doing at least 150 minutes of physical activity a week. Those with SBP >140 mmHg at baseline had a lower risk of dementia compared to those with lower SBP. The risk of incident dementia did not differ by education, sex, BMI, cholesterol, untreated hypertension, and CAIDE dementia risk score.

**TABLE 2:** Incidence of all‐cause dementia in subgroups of interest in the pooled dataset.

Subgroups	Dementia cases	Incidence/100 person‐years (95% CI)	HR (95% CI)
< 75 years	202/2796 (7.2%)	0.93 (0.81–1.06)	
≥ 75 years	284/2374 (9.4%)	1.76 (1.57–1.99)	1.99 (1.66–2.38)
Men	198/2180 (9.0%)	1.24 (1.08–1.42)	
Women	288/2990 (9.6%)	1.32 (1.17–1.48)	1.05 (0.88–1.26)
Low education	118/919 (12.8%)	1.55 (1.29–1.85)	
Medium education	253/2793 (9.0%)	1.20 (1.06–1.36)	0.81 (0.65–1.01)
High education	105/1387 (7.6%)	1.19 (0.99–1.45)	0.88 (0.67–1.15)
*APOE* ε4 non‐carrier	203/3133 (6.5%)	0.85 (0.74–0.97)	
*APOE* ε4 carrier	180/1104 (16.3%)	2.23 (1.93–2.58)	2.72 (2.23–3.33)
MMSE ≥ 26	389/4754 (8.2%)	1.10 (1.00–1.22)	
MMSE < 26	96/410 (23.4%)	3.84 (3.15–4.69)	3.75 (2.99–4.72)
BMI ≤ 30	386/4096 (9.4%)	1.31 (1.19–1.45)	
BMI > 30	98/1065 (9.2%)	1.18 (0.97–1.44)	0.88 (0.71–1.10)
Total cholesterol ≤6.5 mmol/L	397/4173 (9.5%)	1.26 (1.14–1.39)	
Total cholesterol > 6.5 mmol/L	71/696 (10.2%)	1.45 (1.15–1.83)	1.17 (0.91–1.51)
≥ 150 minutes physical activity/week	364/4031 (9.1%)	1.19 (1.08–1.32)	
< 150 minutes physical activity/week	120/1122 (10.7%)	1.66 (1.39–1.99)	1.48 (1.20–1.83)
SBP ≤ 140 mmHg	159/1834 (8.7%)	1.42 (1.21–1.66)	
SBP > 140 mmHg	324/3314 (9.8%)	1.23 (1.10–1.37)	0.78 (0.65–0.95)
No untreated hypertension	341/3701 (9.2%)	1.33 (1.20–1.48)	
Untreated hypertension	144/1457 (9.9%)	1.19 (1.01–1.40)	0.84 (0.69–1.02)
CAIDE < 6	16/332 (4.8%)	0.82 (0.50–1.35)	
CAIDE ≥ 6	430/4391 (9.8%)	1.29 (1.17–1.41)	1.37 (0.83–2.26)

Abbreviation: *APOE*, apolipoprotein E; BMI, body mass index; CAIDE, Cardiovascular Risk Factors, Aging, and Incidence of Dementia; CI, confidence interval; HR, hazard ratio; MMSE, Mini‐Mental State Examination; SBP, systolic blood pressure.

**FIGURE 1:**
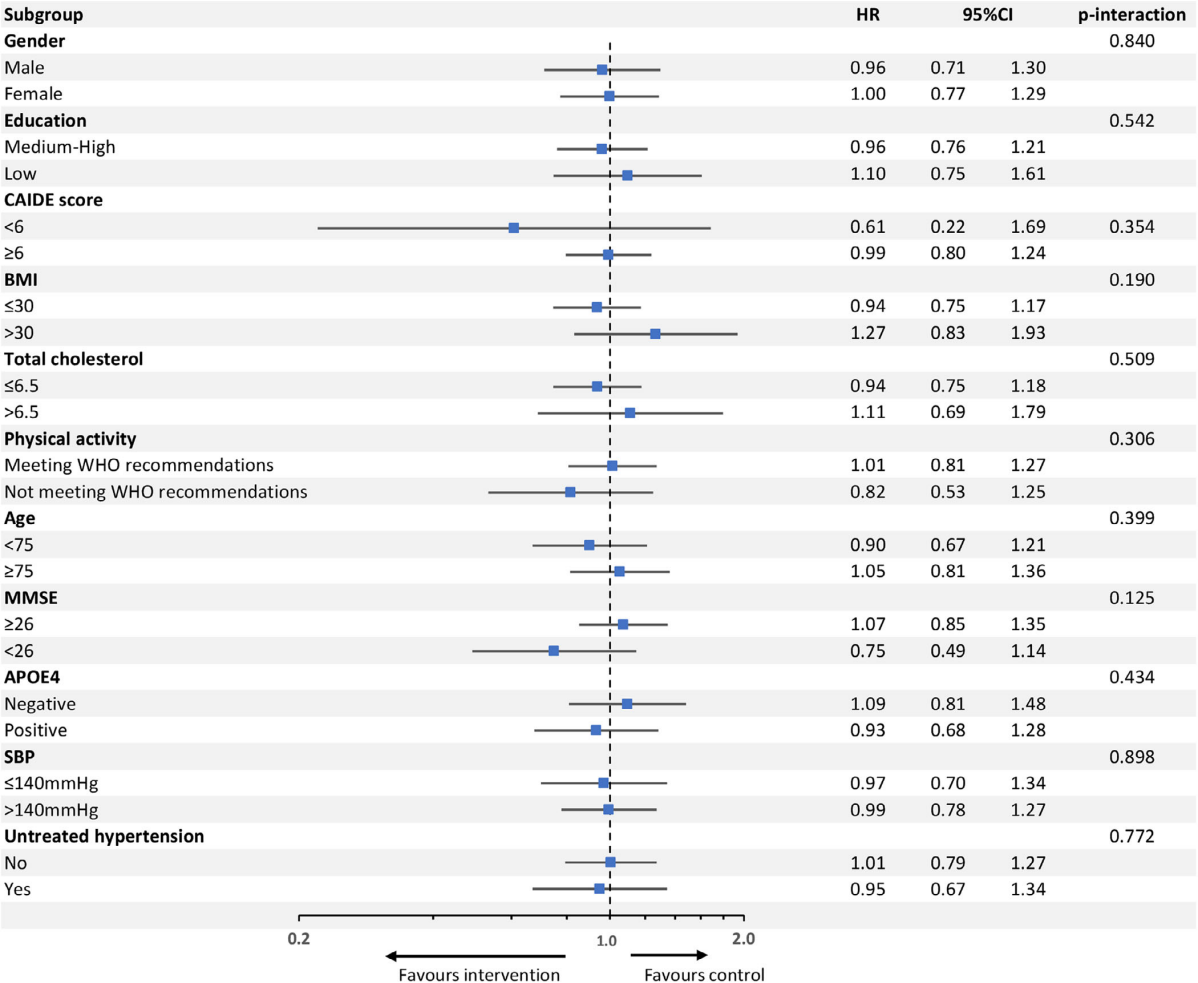
Risk of incident all‐cause dementia in the multidomain intervention versus control group in the pooled dataset across subgroups of interest. Point estimates indicate HRs for incident dementia in the multidomain versus control group within each of the subgroups. Error bars are 95% CIs. Results are unadjusted. *APOE*, apolipoprotein E; BMI, body mass index; CAIDE, Cardiovascular Risk Factors, Aging, and Incidence of Dementia; CI, confidence interval; HR, hazard ratio; MMSE, Mini‐Mental State Examination; SBP, systolic blood pressure; WHO, World Health Organization

In the pooled dataset, there was no effect of multidomain intervention on the risk of all‐cause dementia (HR 0.98, 95% CI 0.80–1.21; see Figure S1 in supporting information for Kaplan–Meier survival curve), and the intervention effect did not differ between the two trials (*P*‐interaction: 0.928). There was also no difference in the effect of the intervention in any of the pre‐specified subgroups. Unadjusted results are shown in Figure 1; there was no substantial change in the results after adjustment for SBP and LDL cholesterol (Table S2 in supporting information). Kaplan–Meier curves can be found in Figures S2–S12 in supporting information. In secondary analyses using AD dementia as the outcome, there was also no difference in the effect of the intervention in any of the pre‐specified subgroups (Table 3). In the data‐driven recursive partitioning analysis, the SIDES algorithm did not detect any subgroup showing a differential intervention effect on incident all‐cause dementia (data not shown).

**TABLE 3:** Risk of incident Alzheimer's disease dementia in the multidomain intervention versus control group in the pooled dataset across subgroups of interest.

	HR	95% CI		*P*‐interaction
**Sex**				0.825
Male	1.01	0.67	1.53	
Female	1.07	0.75	1.52	
**Education**				0.487
Medium–high	1.00	0.73	1.38	
Low	1.23	0.72	2.12	
**CAIDE score**				0.993
< 6	1.04	0.21	5.18	
≥ 6	1.03	0.76	1.40	
**BMI**				0.197
≤ 30	0.99	0.75	1.34	
> 30	1.55	0.81	2.95	
**Total cholesterol**				0.120?
≤ 6.5 mmol/L	1.13	0.83	1.56	
> 6.5 mmol/L	0.64	0.32	1.26	
**Physical activity**				0.402
Meeting WHO recommendations	1.09	0.79	1.51	
Not meeting WHO recommendations	0.86	0.53	1.40	
**Age**				0.609
< 75 years	0.96	0.63	1.48	
≥ 75 years	1.10	0.78	1.56	
**MMSE**				0.519
≥ 26	1.14	0.82	1.58	
< 26	0.94	0.56	1.58	
***APOE* ε4**				0.343
Negative	1.25	0.81	1.93	
Positive	0.95	0.62	1.46	
**SBP**				0.260
≤ 140 mmHg	1.28	0.81	2.02	
> 140 mmHg	0.94	0.67	1.32	
**Untreated hypertension**				0.071
No	1.20	0.87	1.66	
Yes	0.72	0.44	1.18	

Abbreviation: *APOE*, apolipoprotein E; BMI, body mass index; CAIDE, Cardiovascular Risk Factors, Aging, and Incidence of Dementia; CI, confidence interval; HR, hazard ratio; MMSE, Mini‐Mental State Examination; SBP, systolic blood pressure; WHO, World Health Organization.

**FIGURE 2 alz14472-fig-0002:**
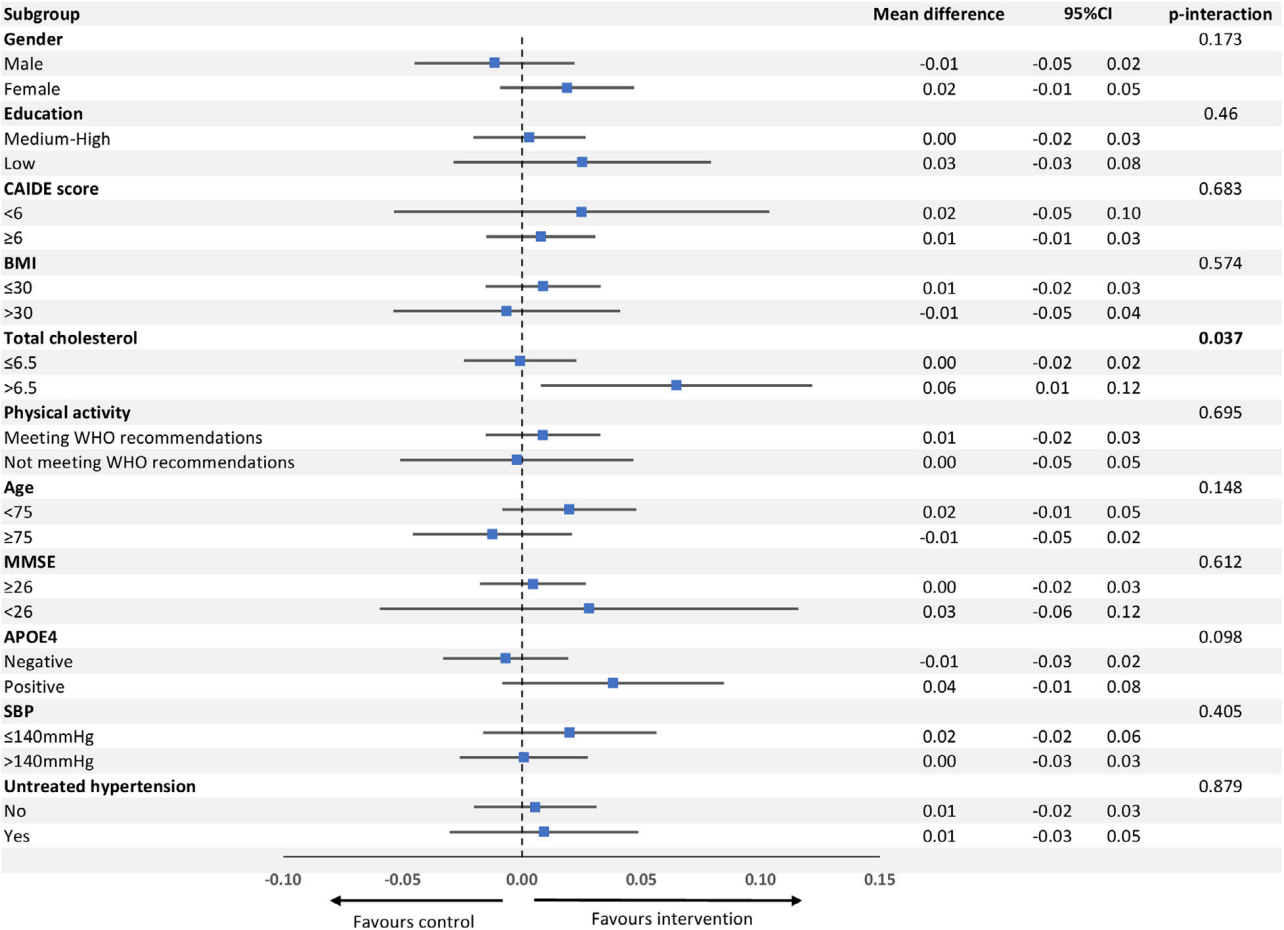
Mean difference in annual rate of MMSE change between multidomain intervention and control groups across subgroups of interest in the pooled dataset. Point estimates are the mean difference in annual rate of MMSE change between multidomain intervention and control groups within each of the subgroups. Error bars are 95% CIs. Results are unadjusted. *APOE*, apolipoprotein E; BMI, body mass index; CAIDE, Cardiovascular Risk Factors, Aging, and Incidence of Dementia; CI, confidence interval; MMSE, Mini‐Mental State Examination; SBP, systolic blood pressure; WHO, World Health Organization

In the pooled dataset, there was no difference in the annual rate of MMSE change between the intervention and control groups (mean difference 0.01 [95% CI −0.02, 0.03]) in the total population, and the intervention effect did not differ between the two trials (*P*‐interaction: 0.733). In the subgroup analyses, there was a significant beneficial effect of the multidomain intervention in individuals with raised baseline total cholesterol (mean difference [95% CI] in annual rate of MMSE change between intervention and control groups 0.06 [0.01, 0.12] vs. 0.00 [−0.02, 0.02] in those with lower cholesterol, *P*‐interaction 0.037). There were no other significant interactions (Figure 2). There was no substantial change in the results after adjustment for SBP and LDL cholesterol (Table S3 in supporting information).

## 4 DISCUSSION

In this pooled analysis of 5205 participants aged 70 and older from the MAPT and preDIVA RCTs, overall, there was no effect of multidomain intervention (involving management of cardiovascular risk factors, and recommendations about diet and exercise, with or without cognitive training) on the risk of incident dementia or the rate of cognitive change, as measured by the MMSE, either in the total population or in pre‐specified subgroups. Furthermore, a recursive partitioning algorithm did not detect any subgroups (based either on single or combinations of risk factors, using any cut‐off for continuously measured factors) in which there was a differential intervention effect.

Although there was a statistically significant intervention effect on MMSE change in individuals with raised baseline total cholesterol, there was no indication of a beneficial intervention effect on all‐cause dementia incidence in this subgroup, and no previous evidence in the literature to support this finding.^22^ One could hypothesize that statin treatment, which could have been initiated during the MAPT and preDIVA trials through the cardiovascular risk factor monitoring part of the interventions, could have had a beneficial effect on cognition, but this was not the primary focus of our interventions, and furthermore, there is relatively little evidence in the literature to support this hypothesis, particularly in older populations.^1^, ^23^, ^24^ In addition, given the high number of subgroups that were tested, this may have been a chance finding.

Based on previous pooled or meta‐analyses assessing multidomain intervention effects on cognitive change during 2 to 4 years of follow‐up,^16^, ^17^ the subgroups in which we might have expected to find the strongest evidence of intervention effects are those defined by a low baseline MMSE score or presence of the *APOE* ε4 genotype. While the highest incidence of dementia in our study was observed in these subgroups, notably in individuals with an MMSE score < 26 at baseline, we did not detect any significant indication of beneficial intervention effects on incident dementia and cognitive change in these subgroups, although there was a non‐significant trend in favor of a possible intervention effect on incident dementia. Despite pooling data from two trials, the low MMSE subgroup contained only 410 participants (8% of the pooled population), and participants with lower MMSE scores were less likely to participate in the extended follow‐up period in MAPT, meaning that some later onset dementia cases were likely missed in this population, reducing statistical power. A 2‐year trial of multidomain behavioral activation, aiming to increase cognitive, physical, and social activity, prevented cognitive and functional decline in 221 individuals with MCI, and ^7^ other trials, often of smaller size, shorter duration, or without clearly defined primary outcome measures, have also provided some evidence of beneficial effects of multidomain interventions on cognitive decline in MCI populations.^25^, ^26^, ^27^, ^28^ However, a recent 18‐month trial of a multidomain intervention in 531 participants with MCI found no effect on a composite cognitive score in its primary analysis.^14^ Further consideration of multidomain interventions for dementia prevention in individuals with signs of early cognitive impairment may still be warranted, with emphasis on increasing adherence which may influence efficacy and may be particularly challenging in this population.^14^, ^29^

Studies evaluating multidomain interventions comprising different intervention components or using different “doses” (intensities), as well as those conducted in younger populations or those with more limited access to primary health care, particularly for the management of cardiovascular risk factors, could provide different results to ours. However, it should be noted that while the window of opportunity for improvement of risk factors may be longer in those with a more unfavorable risk profile, it remains difficult to reach such populations.^30^ Furthermore, alternative clinical or biological risk stratification parameters to those we studied may affect intervention efficacy. For AD, the increasing availability of reliable blood‐based biomarkers could help to refine selection criteria for clinical trials testing interventions expected to be more effective in individuals with a given biomarker profile.^31^ It is unknown to what extent blood‐based biomarkers predict multidomain intervention effects in prevention trials: so far only small, exploratory analyses have been conducted, including in the MAPT trial, and have provided little evidence of effect modification.^14^, ^32^, ^33^ Blood‐based AD biomarkers were not available in preDIVA and thus their effect on intervention efficacy could not be assessed in the current study. While further study of this question could be merited, strong justification would be required before using blood‐based biomarkers in the general population in the absence of cognitive impairment, and the implications of their use would require careful consideration.

Taking into consideration the results of our study and other RCTs,^6^, ^10^, ^17^ the potential impact of multidomain interventions for dementia prevention at the individual level, even among those at greatest risk of dementia, appears modest at best. Multidomain dementia prevention trials have a number of inherent limitations, such as relatively short intervention and follow‐up periods given the gradual onset of the disease, and relatively low‐intensity interventions. Given these limitations, and Geoffrey Rose's classic prevention paradox (“a large number of people at a small risk may give rise to more cases of disease than the small number who are at a high risk”),^34^ a more effective approach might be to channel dementia prevention efforts into population‐level approaches (i.e., altering societal conditions to reduce dementia risk factors or increase exposure to protective factors, for example through environmental and policy measures).^10^, ^35^, ^36^, ^37^

The main strength of our study is that we pooled long‐term follow‐up data on dementia incidence from two large RCTs, providing more power than in an individual study to be able to analyze intervention effects across different subgroups, and more generalizability. However, pooling data from different multidomain dementia prevention trials is complicated by the potential addition of heterogeneity which may arise, in particular, due to a lack of standardization of interventions, target population, and data collection. Indeed, there were differences between the MAPT and preDIVA trials in terms of participant characteristics, outcome measurement, and intervention design. Although both interventions focused on dementia risk factors, and in particular cardiovascular risk factors, preDIVA did not include a cognitive training component, and gave more emphasis to pharmacological treatment than MAPT. Furthermore, the method of delivery, duration, and intensity of the interventions differed between the two trials. Nevertheless, the pooled intervention and control groups were well balanced, and we used appropriate multilevel modelling to account for potential heterogeneity in intervention effects across the two trials (even though statistical interaction tests gave no indication of heterogeneity). Furthermore, the only common measure of cognitive function in the two trials was the MMSE, which is insensitive as an outcome measure, notably in prevention trial settings, thus limiting our ability to detect modest intervention effects in this analysis.[Fig alz14472-fig-0002]

Finally, in the MAPT study, while dementia case‐finding was improved by the thorough cognitive evaluations performed in memory centers for all participants at least annually, the participation rate in the extended follow‐up period was suboptimal, particularly among those most likely to develop dementia, thus limiting statistical power. Nonetheless, non‐participation in this period was not associated with randomization group, thus limiting the impact of attrition bias on our results. As is often the case in prevention trials,^38^, ^39^, ^40^, ^41^ our study population may have been subject to selection bias, which could have affected our results. Indeed, overall our participants appear relatively healthy, for example in terms of BMI and level of physical activity (Table 1). However, the multinational nature of our study, and the use of diverse recruitment methods across the two trials, and in particular the primary care setting of the preDIVA trial, help to increase the generalizability of our findings, at least for settings with similar levels of primary health‐care coverage. Nonetheless, selection bias may have limited the window of opportunity for prevention in our study participants.

In conclusion, we found no convincing evidence to recommend the use of multidomain interventions for the prevention of dementia in older adults aged ≥ 70, either in the general population or subgroups defined by dementia risk factors. Our results are dependent on our study population and definitions of risk factors, but overall, current evidence for the efficacy of multidomain interventions for preventing dementia at the individual level, regardless of age or risk profile, remains scarce, and modest at best. Population‐based approaches to dementia prevention might be more effective.

## CONFLICTS OF INTEREST STATEMENT

Prof. Andrieu reports grants from Occitania Region (No 1901175) and European Regional Development Funds (MP0022856), has received payment/honoraria from Roche, and has served as consultant for Biogen with personal compensation. The other authors report no conflicts of interest. Author disclosures are available in the supporting information.

## CONSENT STATEMENT

All human subjects provided informed consent.

## Supporting information



Supporting Information

Supporting Information

## Data Availability

Individual de‐identified participant data from the MAPT and preDIVA trials are available with immediate effect to academic researchers, after approval of a methodologically sound research proposal by the MAPT/preDIVA data sharing committee and signature of a data access agreement. Enquiries or proposals for MAPT should be directed to nicola.coley@inserm.fr and guyonnet.s@chu‐toulouse.fr, and for preDIVA to m.p.hoevenaarblom@amsterdamumc.nl.

## COLLABORATORS

### CONSORTIA/STUDY GROUP MEMBERS

#### MAPT/IHU HealthAge Open Science group

MAPT Study Group. Principal investigator: Bruno Vellas (Toulouse); Coordination: Sophie Guyonnet; Project leader: Isabelle Carrié; CRA: Lauréane Brigitte; Investigators: Catherine Faisant, Françoise Lala, Julien Delrieu, Hélène Villars; Psychologists: Emeline Combrouze, Carole Badufle, Audrey Zueras; Methodology, statistical analysis, and data management: Sandrine Andrieu, Christelle Cantet, Christophe Morin; Multidomain group: Gabor Abellan Van Kan, Charlotte Dupuy, Yves Rolland (physical and nutritional components), Céline Caillaud, Pierre‐Jean Ousset (cognitive component), Françoise Lala (preventive consultation). The cognitive component was designed in collaboration with Sherry Willis from the University of Seattle, and Sylvie Belleville, Brigitte Gilbert, and Francine Fontaine from the University of Montreal.

Co‐Investigators in associated centers: Jean‐François Dartigues, Isabelle Marcet, Fleur Delva, Alexandra Foubert, Sandrine Cerda (Bordeaux); Marie‐Noëlle‐Cuffi, Corinne Costes (Castres); Olivier Rouaud, Patrick Manckoundia, Valérie Quipourt, Sophie Marilier, Evelyne Franon (Dijon); Lawrence Bories, Marie‐Laure Pader, Marie‐France Basset, Bruno Lapoujade, Valérie Faure, Michael Li Yung Tong, Christine Malick‐Loiseau, Evelyne Cazaban‐Campistron (Foix); Françoise Desclaux, Colette Blatge (Lavaur); Thierry Dantoine, Cécile Laubarie‐Mouret, Isabelle Saulnier, Jean‐Pierre Clément, Marie‐Agnès Picat, Laurence Bernard‐Bourzeix, Stéphanie Willebois, Iléana Désormais, Noëlle Cardinaud (Limoges); Marc Bonnefoy, Pierre Livet, Pascale Rebaudet, Claire Gédéon, Catherine Burdet, Flavien Terracol (Lyon), Alain Pesce, Stéphanie Roth, Sylvie Chaillou, Sandrine Louchart (Monaco); Kristelle Sudres, Nicolas Lebrun, Nadège Barro‐Belaygues (Montauban); Jacques Touchon, Karim Bennys, Audrey Gabelle, Aurélia Romano, Lynda Touati, Cécilia Marelli, Cécile Pays (Montpellier); Philippe Robert, Franck Le Duff, Claire Gervais, Sébastien Gonfrier (Nice); Yannick Gasnier and Serge Bordes, Danièle Begorre, Christian Carpuat, Khaled Khales, Jean‐François Lefebvre, Samira Misbah El Idrissi, Pierre Skolil, Jean‐Pierre Salles (Tarbes).

MRI group: Carole Dufouil (Bordeaux), Stéphane Lehéricy, Marie Chupin, Jean‐François Mangin, Ali Bouhayia (Paris); Michèle Allard (Bordeaux); Frédéric Ricolfi (Dijon); Dominique Dubois (Foix); Marie Paule Bonceour Martel (Limoges); François Cotton (Lyon); Alain Bonafé (Montpellier); Stéphane Chanalet (Nice); Françoise Hugon (Tarbes); Fabrice Bonneville, Christophe Cognard, François Chollet (Toulouse).

PET scans group: Pierre Payoux, Thierry Voisin, Julien Delrieu, Sophie Peiffer, Anne Hitzel, (Toulouse); Michèle Allard (Bordeaux); Michel Zanca (Montpellier); Jacques Monteil (Limoges); Jacques Darcourt (Nice).

Medico‐economics group: Laurent Molinier, Hélène Derumeaux, Nadège Costa (Toulouse).

Biological sample collection: Bertrand Perret, Claire Vinel, Sylvie Caspar‐Bauguil (Toulouse).

Safety management: Pascale Olivier‐Abbal

IHU HealthAge Open Science Group: Nicola Coley, Sandrine Andrieu, Christelle Cantet, Sophie Guyonnet

PreDIVA

The members of the preDIVA group are: Eric P. Moll van Charante, Edo Richard, Lisa S. Eurelings, Jan‐Willem van Dalen, Suzanne A. Ligthart, Emma F. van Bussel, Marieke P. Hoevenaar‐Blom, Marinus Vermeulen, Willem A. van Gool (Amsterdam Medical Centre, Amsterdam, the Netherlands). We are indebted to all practice nurses delivering the intervention and all general practitioners involved in the care for the participants, including the “Zorggroep Almere.” We particularly thank our project manager C.E. Miedema for her outstanding role in coordinating the trial. We acknowledge the efforts of the interim committee members (Dr. A. de Craen†, Prof. N. de Wit, and Prof. J. Stam). We thank the members of the independent outcome adjudication committee.
