# Supplementary material for: Searching for responders to multidomain dementia prevention in late life: A pooled analysis of individual participant data from the MAPT and preDIVA trials
Source: Alzheimers Dement. 2025 Jan 17;21(2):e14472. doi: 10.1002/alz.14472 (PMC11848180; doi:10.1002/alz.14472)

**Supplementary material**

**Supplementary Table 1. Baseline characteristics of MAPT participants who did and did not participate in the extended observational follow-up period**

|  | **Did not participate in extended follow-up**  **(N=697)** | **Participated in extended follow-up**  **(N=982)** | **p** |
| --- | --- | --- | --- |
| Age (y), median (IQR) | 76 (73-80) | 74 (71-77) | **<0.001** |
| MMSE, median (IQR) | 28 (27-29) | 29 (28-29) | **<0.001** |
| CAIDE, mean (SD) | 7.5 (1.8) | 7.2 (1.9) | **0.007** |
| BMI (kg/m²), mean (SD) | 26.2 (4.2) | 26.0 (4.0) | 0.339 |
| SBP (mmHg), mean (SD) | 141.1 (20.0) | 140.9 (19.5) | 0.887 |
| LDL cholesterol (mmol/l), mean (SD) | 3.2 (0.91) | 3.3 (0.92) | 0.072 |
| Total cholesterol (mmol/l), mean (SD) | 5.6 (1.0) | 5.7 (1.1) | 0.056 |
| Female, n(%) | 434 (62.3%) | 653 (66.5%) | 0.074 |
| Education, n(%)  Low  Medium  High | 42 (6.2%)  373 (55.4%)  258 (38.3%) | 43 (4.4%)  466 (48.0%)  461 (47.5%) | **0.001** |
| Diabetes, n(%) | 78 (11.2%) | 71 (7.2%) | **0.005** |
| Stroke, n(%) | 41 (5.9%) | 52 (5.3%) | 0.603 |
| Heart disease, n(%) | 72 (10.3%) | 93 (9.5%) | 0.558 |
| ≥150 min physical activity/wk, n(%) | 489 (70.2%) | 722 (73.5%) | 0.130 |
| APOE Ɛ4, n(%) | 115 (26.7%) | 184 (21.2%) | **0.026** |
| Untreated hypertension | 110 (15.9%) | 182 (18.6%) | 0.155 |
| Intervention group, n(%)  Multidomain  Non-multidomain | 349 (50.1%)  348 (49.9%) | 488 (49.7%)  494 (50.3%) |  |

**Supplementary Table 2. Adjusted risk of incident all-cause dementia in the multidomain intervention versus control group in the pooled dataset across subgroups of interest.**

|  | **HR** | **95%CI** | | **p-interaction** |
| --- | --- | --- | --- | --- |
| **Gender** |  |  |  | 0.828 |
| Male | 0.94 | 0.69 | 1.29 |  |
| Female | 0.98 | 0.76 | 1.28 |  |
| **Education** |  |  |  | 0.510 |
| Medium-High | 0.94 | 0.74 | 1.21 |  |
| Low | 1.09 | 0.74 | 1.62 |  |
| **CAIDE score** |  |  |  | 0.340 |
| <6 | 0.61 | 0.22 | 1.69 |  |
| ≥6 | 1.01 | 0.81 | 1.26 |  |
| **BMI** |  |  |  | 0.176 |
| ≤30 | 0.92 | 0.73 | 1.16 |  |
| >30 | 1.27 | 0.82 | 1.98 |  |
| **Total cholesterol** |  |  |  | 0.553 |
| ≤6.5 mmol/l | 0.94 | 0.75 | 1.18 |  |
| >6.5 mmol/l | 1.10 | 0.68 | 1.78 |  |
| **Physical activity** |  |  |  | 0.430 |
| Meeting WHO recommendations | 0.84 | 0.57 | 1.24 |  |
| Not meeting WHO recommendations | 1.00 | 0.78 | 1.28 |  |
| **Age** |  |  |  | 0.311 |
| <75y | 0.87 | 0.64 | 1.18 |  |
| ≥75y | 1.05 | 0.81 | 1.38 |  |
| **MMSE** |  |  |  | 0.195 |
| ≥26 | 1.04 | 0.83 | 1.33 |  |
| <26 | 0.77 | 0.50 | 1.18 |  |
| **APOE4** |  |  |  | 0.435 |
| Negative | 1.06 | 0.78 | 1.45 |  |
| Positive | 0.90 | 0.65 | 1.26 |  |
| **SBP** |  |  |  | 0.713 |
| ≤140mmHg | 0.92 | 0.66 | 1.29 |  |
| >140mmHg | 0.99 | 0.77 | 1.28 |  |
| **Untreated hypertension** |  |  |  | 0.857 |
| No | 0.98 | 0.77 | 1.26 |  |
| Yes | 0.95 | 0.66 | 1.35 |  |

Models were adjusted for baseline systolic blood pressure and LDL cholesterol

**Supplementary Table 3. Adjusted mean difference in annual rate of MMSE change between multidomain intervention and control groups across subgroups of interest in the pooled dataset.**

| **Subgroup** | **Mean difference** | **95%CI** | | **p-interaction** |
| --- | --- | --- | --- | --- |
| **Gender** |  |  |  | 0.236 |
| Male | -0.01 | -0.04 | 0.03 |  |
| Female | 0.02 | -0.01 | 0.05 |  |
| **Education** |  |  |  | 0.503 |
| Medium-High | 0.01 | -0.02 | 0.03 |  |
| Low | 0.03 | -0.03 | 0.08 |  |
| **CAIDE score** |  |  |  | 0.78 |
| <6 | 0.02 | -0.06 | 0.10 |  |
| ≥6 | 0.01 | -0.02 | 0.03 |  |
| **BMI** |  |  |  | 0.777 |
| ≤30 | 0.01 | -0.01 | 0.04 |  |
| >30 | -0.01 | -0.05 | 0.04 |  |
| **Total cholesterol** |  |  |  | **0.040** |
| ≤6.5 mmol/l | 0.00 | -0.02 | 0.02 |  |
| >6.5 mmol/l | 0.07 | 0.01 | 0.12 |  |
| **Physical activity** |  |  |  | 0.595 |
| Meeting WHO recommendations | 0.01 | -0.01 | 0.04 |  |
| Not meeting WHO recommendations | 0.00 | -0.05 | 0.05 |  |
| **Age** |  |  |  | 0.099 |
| <75y | 0.03 | 0.00 | 0.05 |  |
| ≥75y | -0.01 | -0.05 | 0.02 |  |
| **MMSE** |  |  |  | 0.264 |
| ≥26 | 0.01 | -0.02 | 0.03 |  |
| <26 | 0.06 | -0.03 | 0.15 |  |
| **APOE4** |  |  |  | 0.092 |
| Negative | 0.00 | -0.03 | 0.02 |  |
| Positive | 0.04 | -0.01 | 0.09 |  |
| **SBP** |  |  |  | 0.292 |
| ≤140mmHg | 0.03 | -0.01 | 0.06 |  |
| >140mmHg | 0.00 | -0.03 | 0.03 |  |
| **Untreated hypertension** |  |  |  | 0.806 |
| No | 0.01 | -0.02 | 0.03 |  |
| Yes | 0.01 | -0.03 | 0.05 |  |

Models were adjusted for baseline systolic blood pressure and LDL cholesterol

**Supplementary Figure 1. Kaplan-Meier survival curve showing the incidence of all-cause dementia by randomisation group in the pooled dataset**


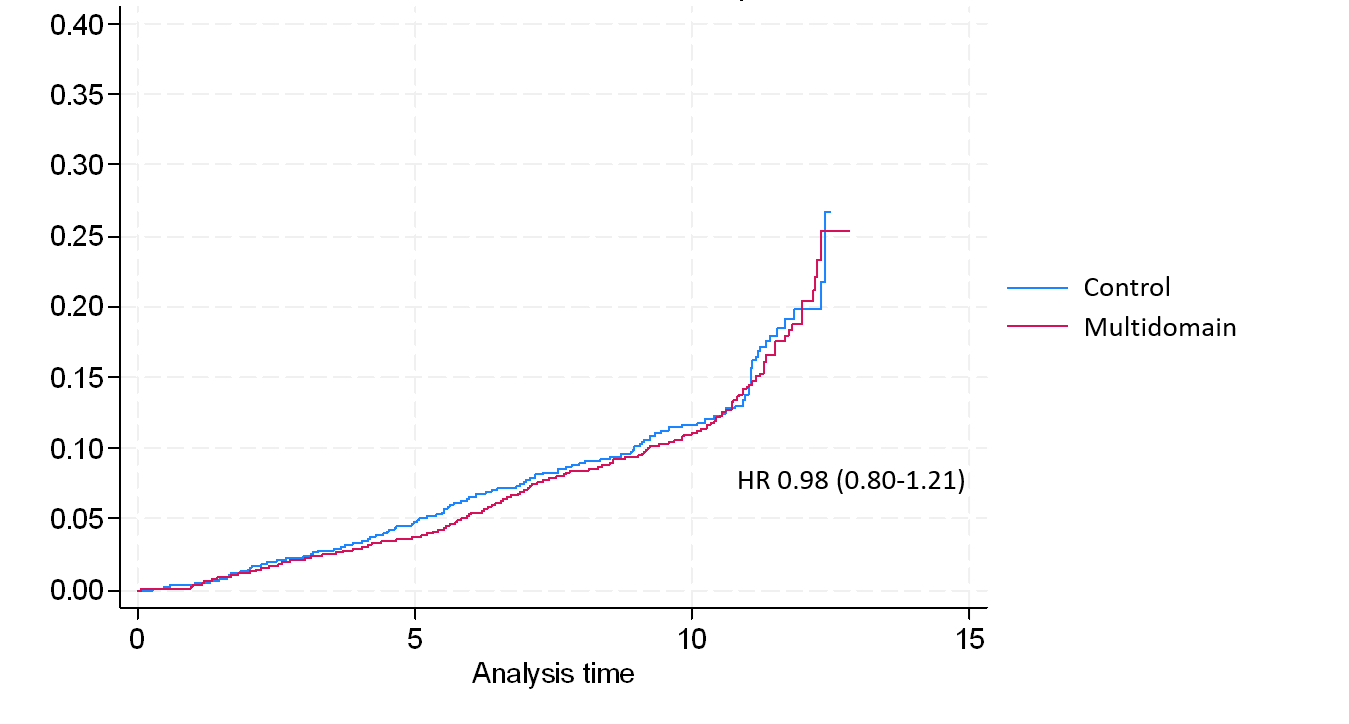


**Supplementary Figure 2. Kaplan-Meier survival curve showing the incidence of all-cause dementia by gender and randomisation group in the pooled dataset**


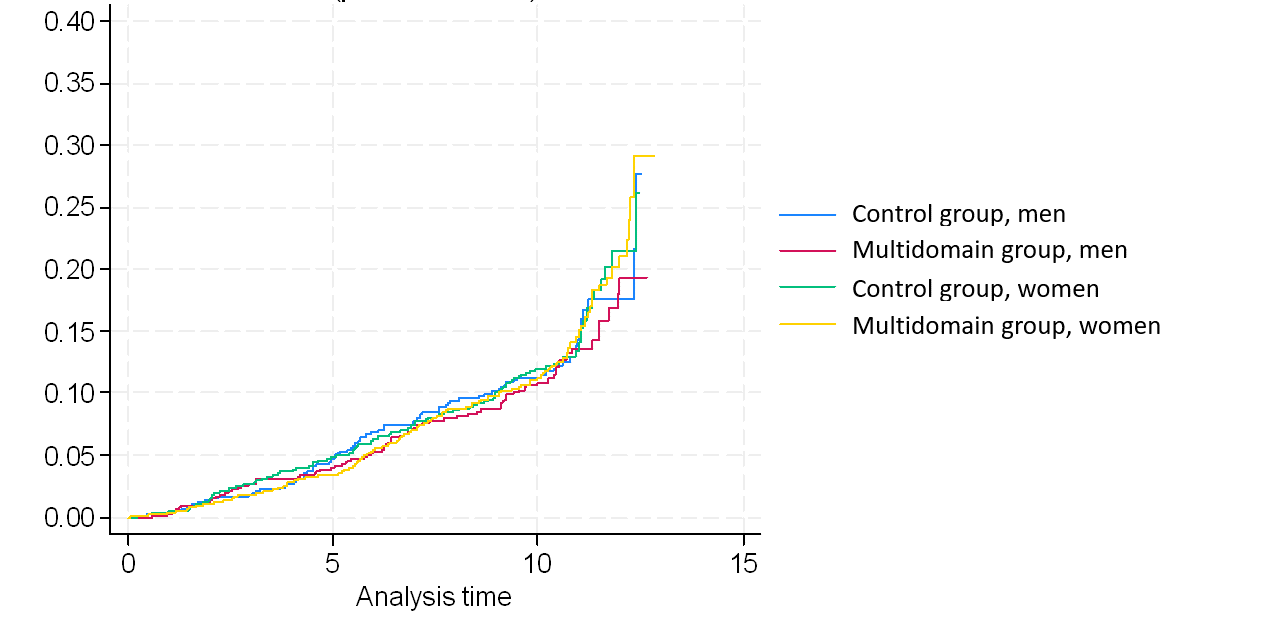


**Supplementary Figure 3. Kaplan-Meier survival curve showing the incidence of all-cause dementia by education and randomisation group in the pooled dataset**


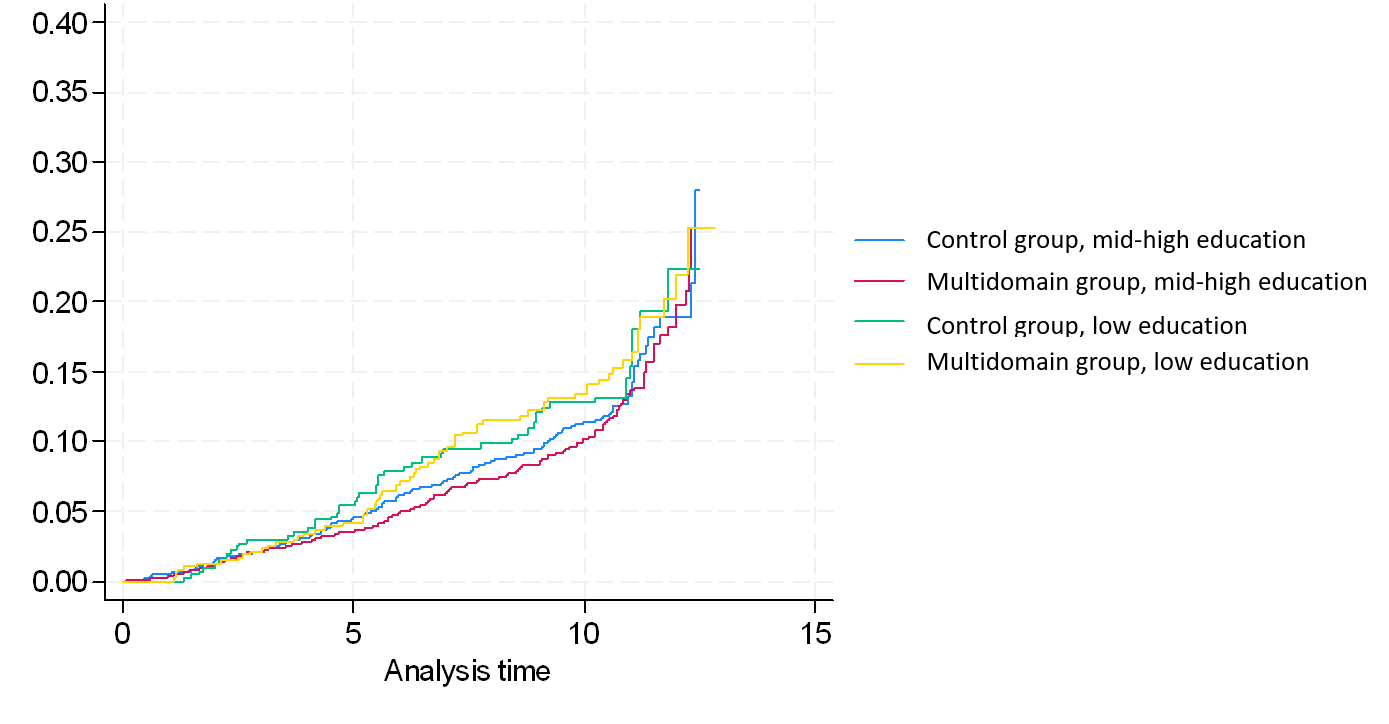


**Supplementary Figure 4. Kaplan-Meier survival curve showing the incidence of all-cause dementia by baseline CAIDE score and randomisation group in the pooled dataset**


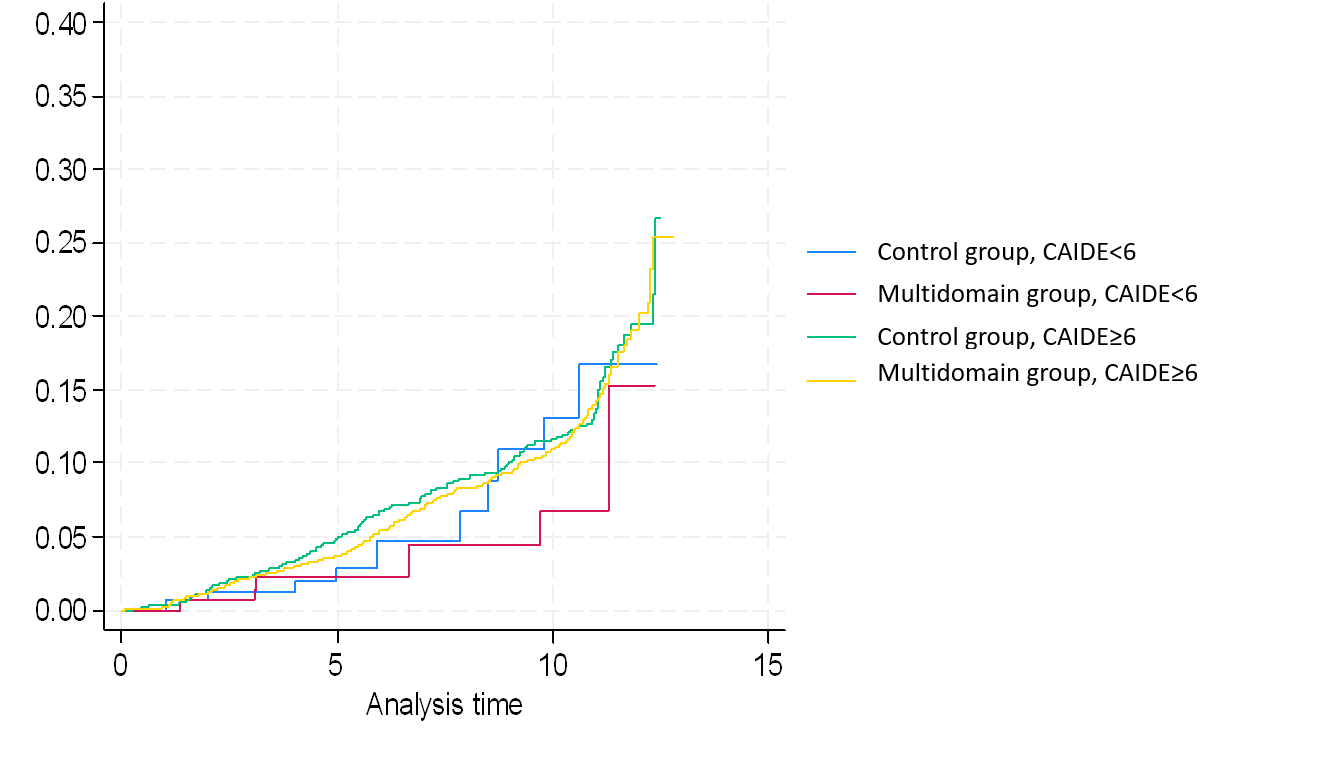


**Supplementary Figure 5. Kaplan-Meier survival curve showing the incidence of all-cause dementia by baseline BMI and randomisation group in the pooled dataset**


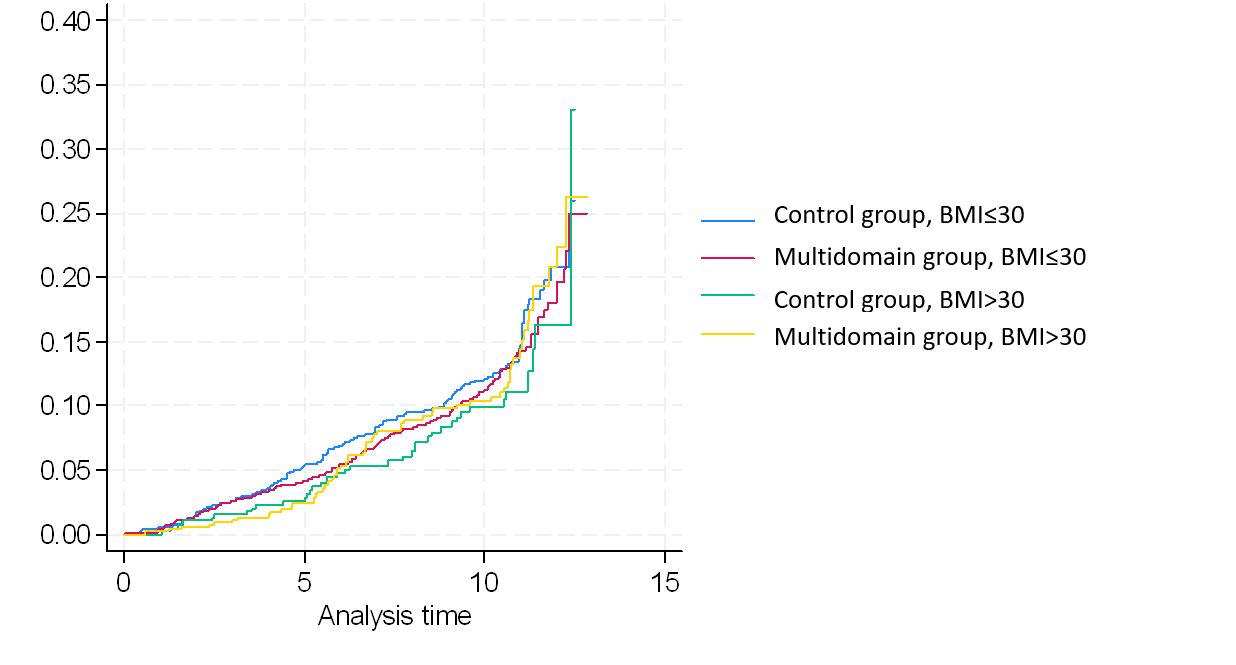


**Supplementary Figure 6. Kaplan-Meier survival curve showing the incidence of all-cause dementia by baseline total cholesterol and randomisation group in the pooled dataset**


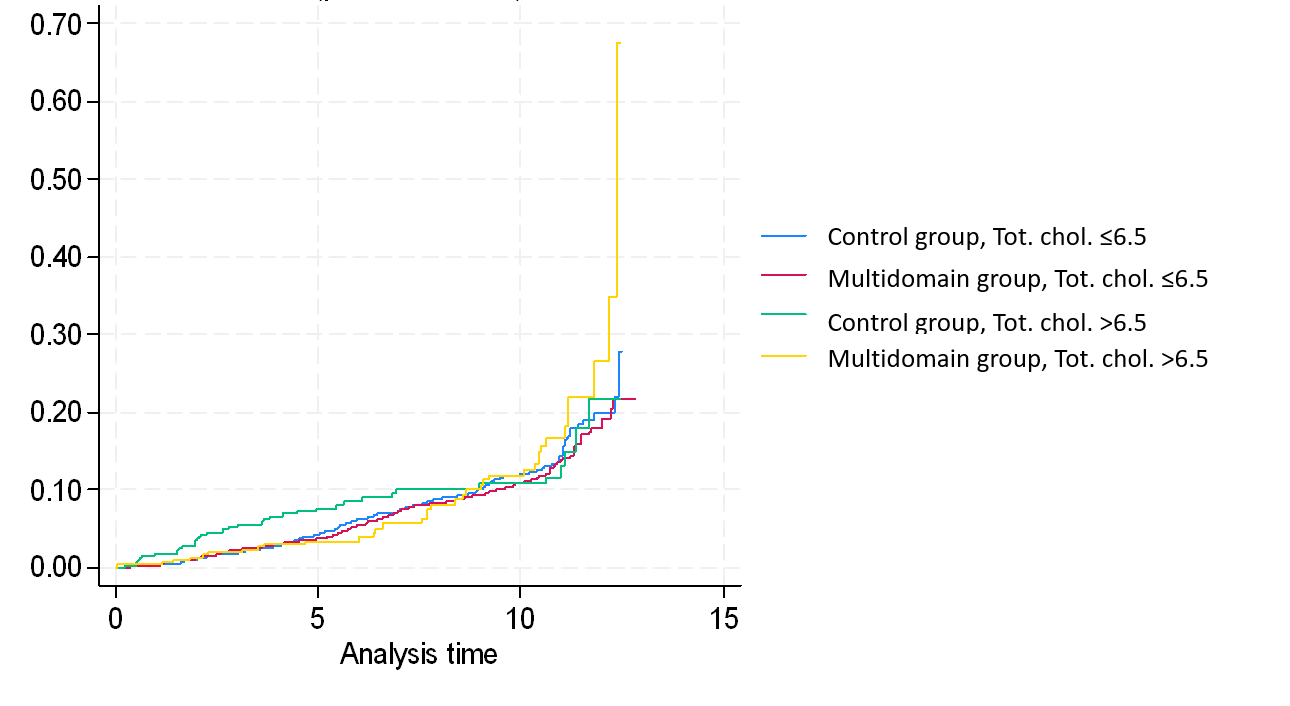


**Supplementary Figure 7. Kaplan-Meier survival curve showing the incidence of all-cause dementia by baseline physical activity and randomisation group in the pooled dataset**


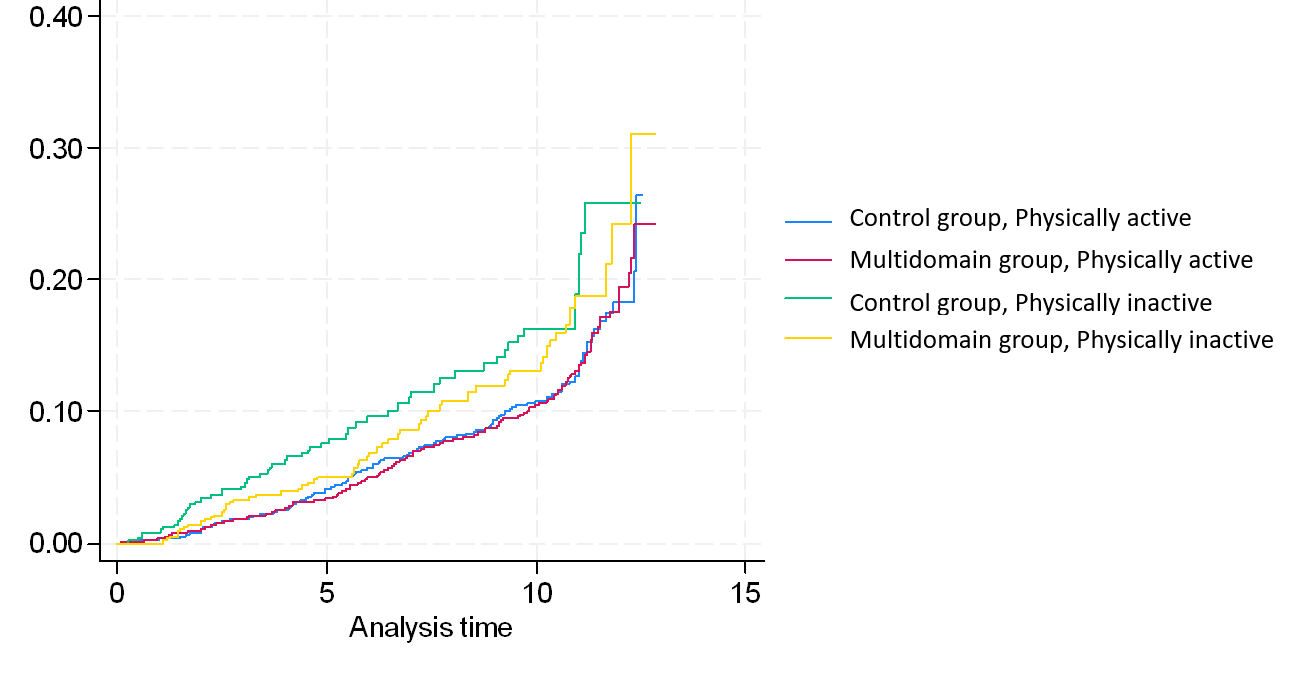


**Supplementary Figure 8. Kaplan-Meier survival curve showing the incidence of all-cause dementia by baseline MMSE score and randomisation group in the pooled dataset**


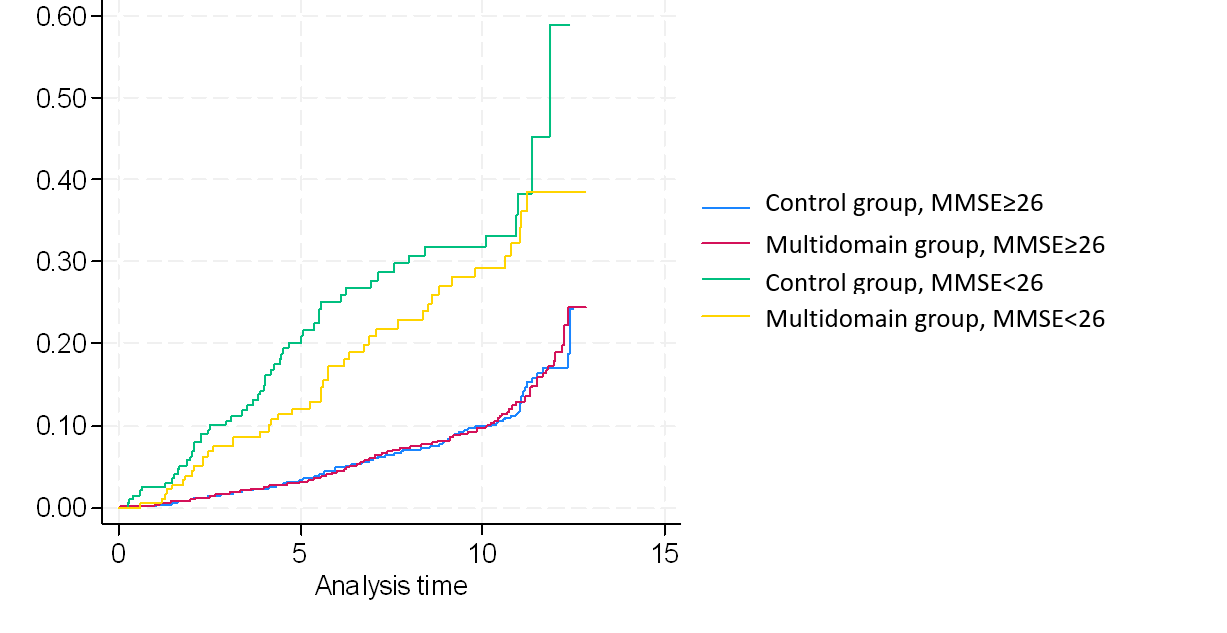


**Supplementary Figure 9. Kaplan-Meier survival curve showing the incidence of all-cause dementia by baseline age and randomisation group in the pooled dataset**


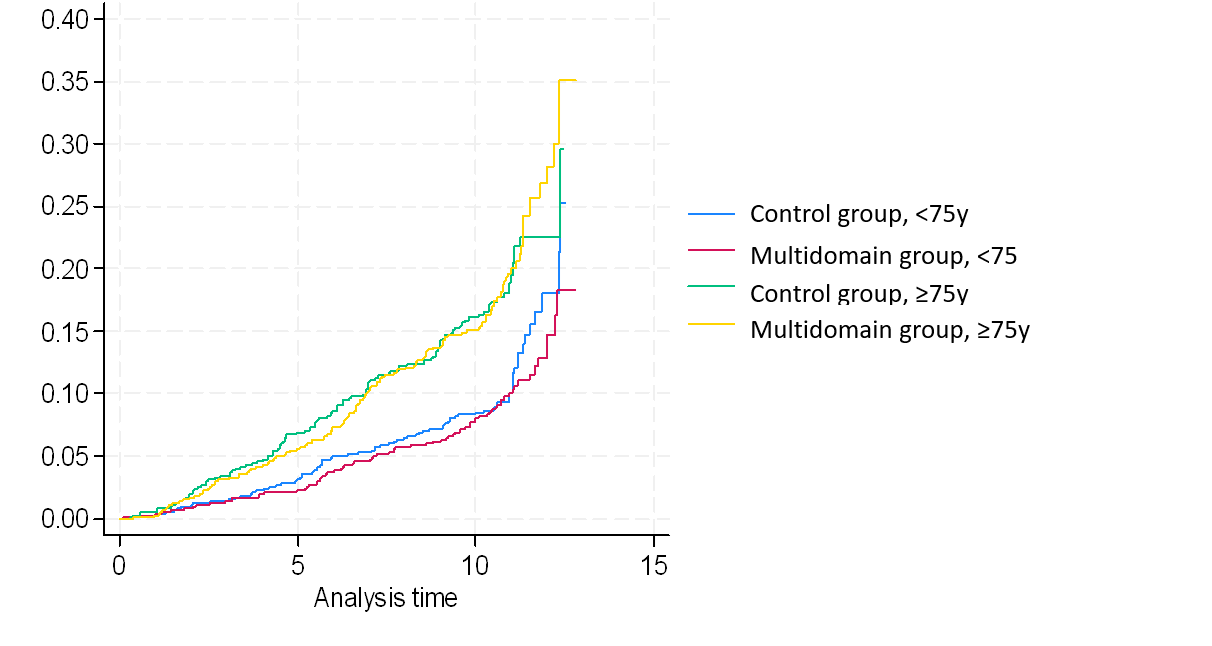


**Supplementary Figure 10. Kaplan-Meier survival curve showing the incidence of all-cause dementia by APOE genotype and randomisation group in the pooled dataset**


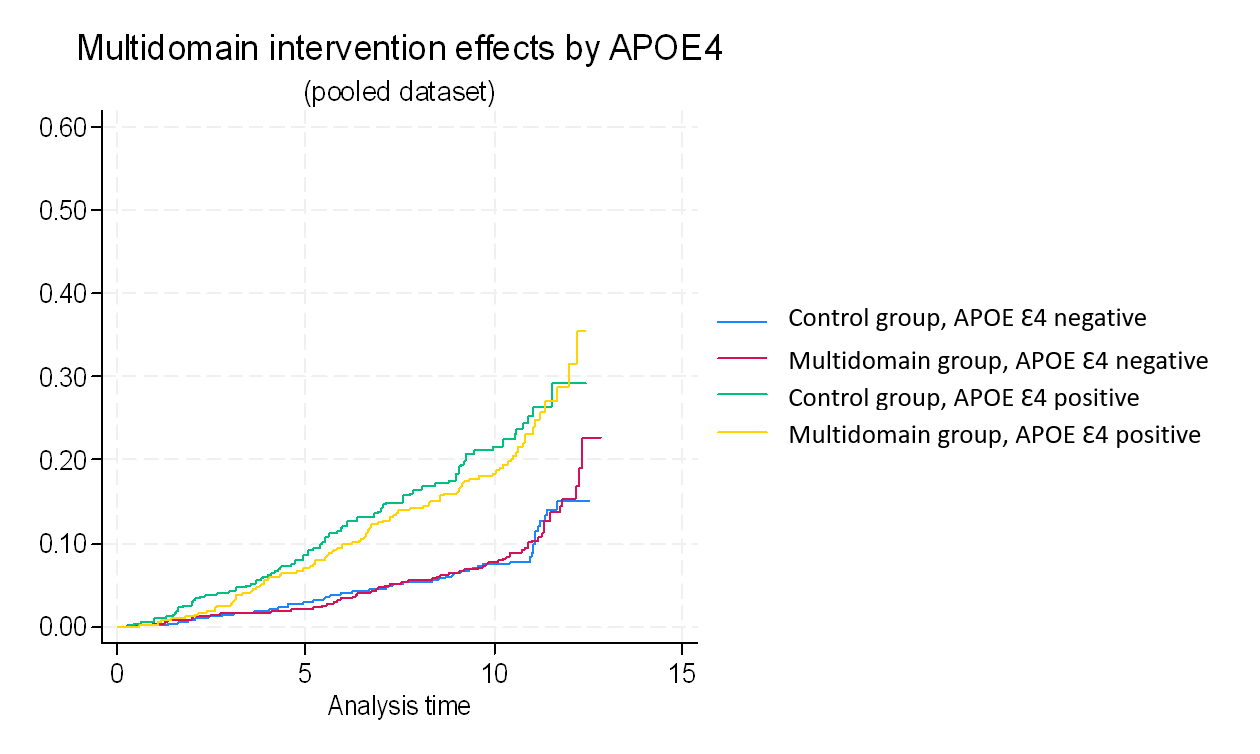


**Supplementary Figure 11. Kaplan-Meier survival curve showing the incidence of all-cause dementia by baseline systolic blood pressure and randomisation group in the pooled dataset**


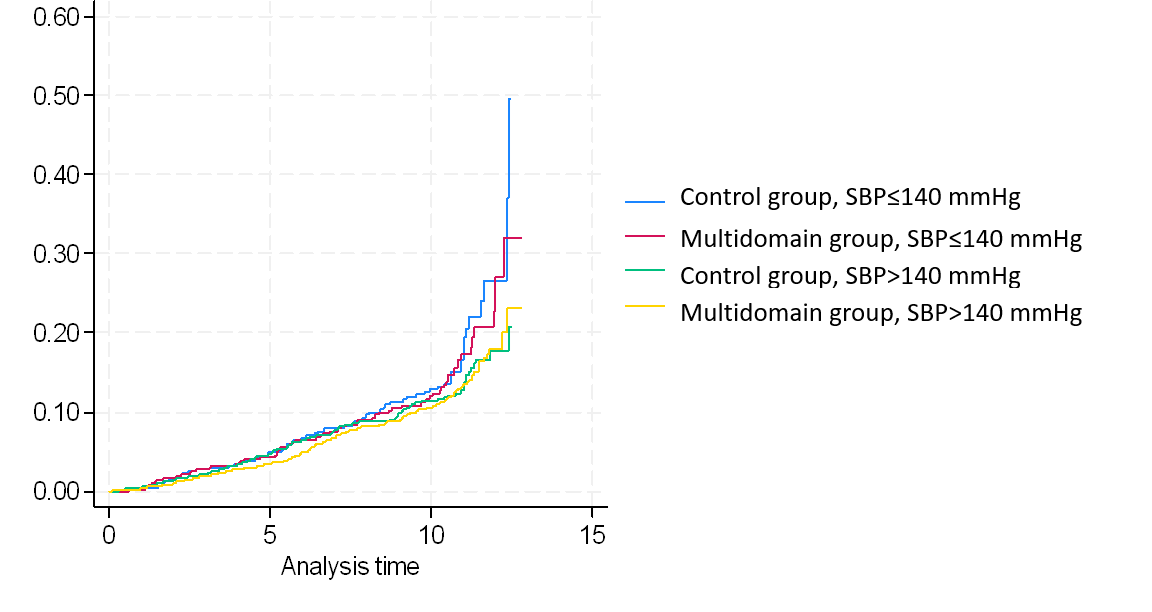


**Supplementary Figure 12. Kaplan-Meier survival curve showing the incidence of all-cause dementia by presence of untreated hypertension at baseline and randomisation group in the pooled dataset**


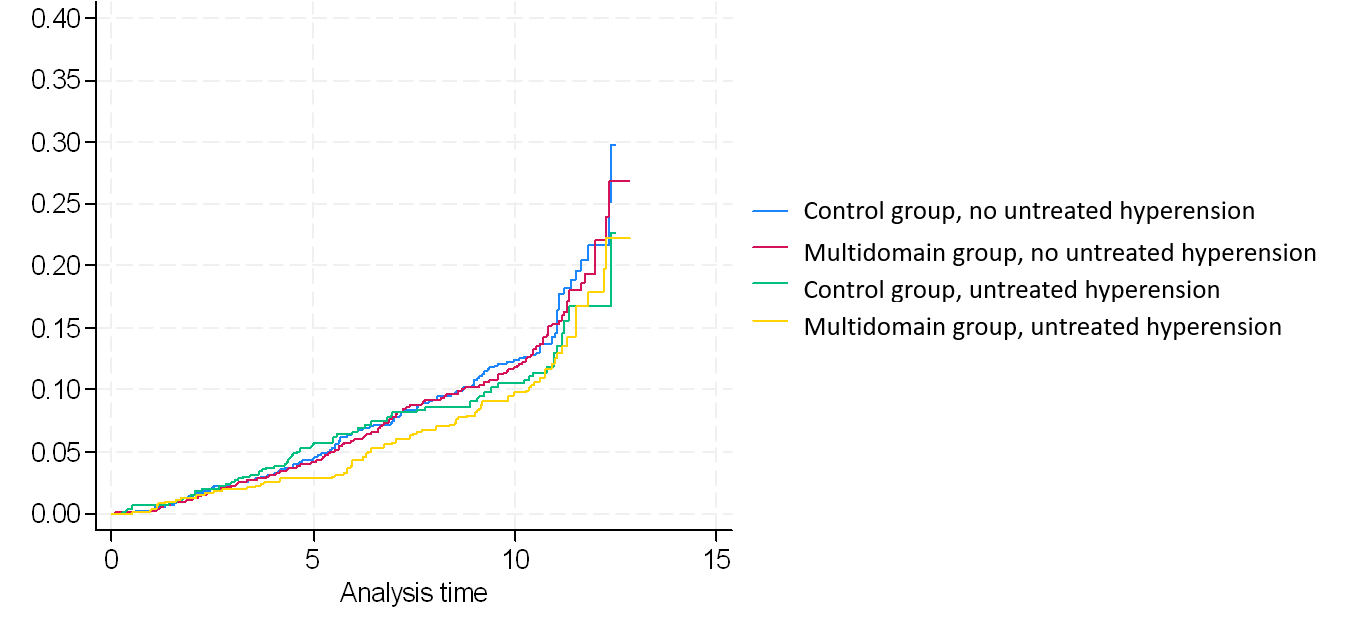

Supplement: Supplementary file 1 — Supporting Information [file ALZ-21-e14472-s001.docx]
